# Alpha-1 Antitrypsin PiMZ Heterozygosity in the Setting of Alcohol-Associated Cirrhosis: A Case Report

**DOI:** 10.7759/cureus.115864

**Published:** 2026-09-06

**Authors:** Nimah Ather, Didi Mwengela, John Blakey

**Affiliations:** 1 Department of Medicine, Division of Digestive Diseases, University of California, Los Angeles, Torrance, USA; 2 Department of Pathology, Torrance Memorial Medical Center, Torrance, USA

**Keywords:** alpha-1-antitrypsin deficiency, clinical heterozygosity, cryptogenic cirrhosis, profound hyponatremia, transjugular liver biopsy

## Abstract

Alpha-1 antitrypsin deficiency (AATD) is classically associated with the PiZZ genotype, while PiMZ heterozygosity has historically been considered a benign carrier state. Emerging evidence, however, suggests PiMZ status functions as a clinically significant cofactor for liver fibrosis when combined with metabolic or environmental stressors such as alcohol use. We present the case of a 57-year-old woman with a diagnosis of cirrhosis of unclear etiology and a remote history of alcohol use, who was admitted with jaundice and profound hyponatremia. Workup for viral, autoimmune, and hereditary hemochromatosis etiologies was unrevealing. Transjugular liver biopsy demonstrated periodic acid-Schiff-positive, diastase-resistant (PAS-D) intrahepatocytic globules with mild siderosis; subsequent testing revealed a low serum alpha-1 antitrypsin (A1AT) level and PiMZ genotype. This case illustrates the diagnostic value of liver biopsy in identifying AATD-pattern globules and prompting confirmatory serum and genotypic testing. Although this patient's serum AAT was notably reduced - a diagnostically helpful but not universal finding, as many PiMZ carriers have levels near the normal range - the case underscores that biopsy and genotyping remain essential for diagnosis regardless of serum AAT level. It further reflects the growing recognition that PiMZ heterozygosity, particularly in the presence of alcohol exposure, may meaningfully contribute to progression toward decompensated cirrhosis.

## Introduction

Alpha-1 antitrypsin deficiency (AATD) is an inherited disorder caused by mutations in the SERPINA1 gene, resulting in hepatic accumulation of misfolded A1AT protein that appears histologically as periodic acid-Schiff-positive, diastase-resistant (PAS-D) globules [1]. While the PiZZ homozygous genotype is well established as a cause of clinically significant liver disease, PiMZ heterozygosity has traditionally been considered a benign carrier state [2]. This view has shifted, with PiMZ status now recognized as a cofactor for liver fibrosis in the presence of concurrent risk factors such as alcohol use [3]. Importantly, serum AAT levels alone cannot reliably exclude clinically relevant heterozygosity; hepatic protein aggregation in PiMZ individuals correlates with advanced fibrosis and reduced transplant-free survival independent of serum concentrations [4], raising the possibility that PiMZ-associated liver disease is under-recognized in patients with cirrhosis attributed to more common etiologies. We report a case of decompensated cirrhosis with severe hyponatremia in which biopsy findings prompted genotyping and identification of PiMZ heterozygosity, highlighting the diagnostic value of this workup in cirrhosis of uncertain etiology.

## Case presentation

A 57-year-old female with a nine-month history of cirrhosis of unclear etiology presented to the hospital with progressive weakness and jaundice. The patient was seeing doctors as an outpatient regarding her cirrhosis. There was concern that alcohol was a component, as she did report daily alcohol use from 2012 to 2023, with roughly three years of abstinence before admission. She denied a family history of liver disease. Home medications included furosemide 40 mg and spironolactone 100 mg daily. Her daughter reported significant edema in preceding weeks and large-volume water intake.

On examination, she was jaundiced with scattered spider angiomata, without ascites, asterixis, or clinical encephalopathy. Admission laboratories revealed profound hyponatremia (sodium 103 mEq/L), prompting intensive care admission, with a total bilirubin of 21.3 mg/dL, international normalized ratio (INR) 2.1, and creatinine 0.51 mg/dL, corresponding to a MELD-Na of 32. Given the severity of the hyponatremia and its association with mortality in cirrhosis, biopsy was deferred pending sodium correction.

Prior outpatient evaluation showed negative viral hepatitis serologies. Iron studies and immunoglobulin G were notable, as detailed in Table 1. Genetic testing was sent for hereditary hemochromatosis, and she was found to be HFE C282Y/H63D homozygous wild-type, making hemochromatosis an unlikely diagnosis. ANA and the actin (smooth muscle) antibody returned negative (Table 1).

**Table 1:** Key laboratory investigations.

Laboratory Test	Patient Value	Reference Range
Sodium (admission)	103 mEq/L	135-145 mEq/L
Total bilirubin	21.3 mg/dL	0.1-1.2 mg/dL
Direct bilirubin	9.7 mg/dL	0.0-0.3 mg/dL
Aspartate aminotransferase (AST)	78 U/L	10-40 U/L
Alanine aminotransferase (ALT)	36 U/L	7-56 U/L
Alkaline phosphatase	179 U/L	44-147 U/L
Albumin	2.9 g/dL	3.5-5.0 g/dL
International normalized ratio (INR)	2.1	0.8-1.1
Creatinine	0.51 mg/dL	0.6-1.1 mg/dL
Platelet count	105 ×10⁹/L	150-450 ×10⁹/L
Iron	232 µg/dL	50-170 µg/dL
Transferrin saturation	97%	20-50%
Ferritin	885 ng/mL	10-200 ng/mL
Immunoglobulin G	2292 mg/dL	700-1600 mg/dL
Actin (smooth muscle) antibody	19 units (negative)	0-19 units
Serum alpha-1 antitrypsin (A1AT)	67 mg/dL	100-190 mg/dL

Hospital course 

The patient was managed with hypertonic saline and fluid restriction under nephrology guidance. Sodium improved from 103 to 133 mEq/L over nine days, with a maximum 24-hour increase of 8 mEq/L. This is within the 8 mEq/L per 24 hours and 14 mEq/L per 48 hours generally considered safe for patients at high risk of osmotic demyelination syndrome, including those with chronic liver disease and heavy alcohol use [5]. No neurologic complications occurred during the admission.

Repeat inpatient serologies showed a negative anti-smooth muscle antibody and an equivocal ANA. Contrast-enhanced MRI with MRCP demonstrated gallstones without ductal dilatation, a partially nodular liver contour consistent with cirrhosis, no focal lesion, a spleen at the upper limit of normal (13.2 cm), and trace ascites. The original images were unavailable for inclusion.

On hospital day eight, with sodium at 125 mEq/L, transjugular liver biopsy was performed; this route was chosen given thrombocytopenia (platelets 105 ×10⁹/L) and coagulopathy (INR 2.1). Histology demonstrated cirrhosis with bridging fibrosis (Figure 1) and PAS-D-positive cytoplasmic granules within hepatocytes (Figure 2), with mild siderosis, minimal interface activity, only a minimal plasma cell component, and no granulomata or onion-skin fibrosis. The pathologist recommended correlation with A1AT level and phenotype. Serum A1AT returned low at 67 mg/dL (Table 1), with Pi genotyping demonstrating an MZ heterozygous pattern.

**Figure 1:**
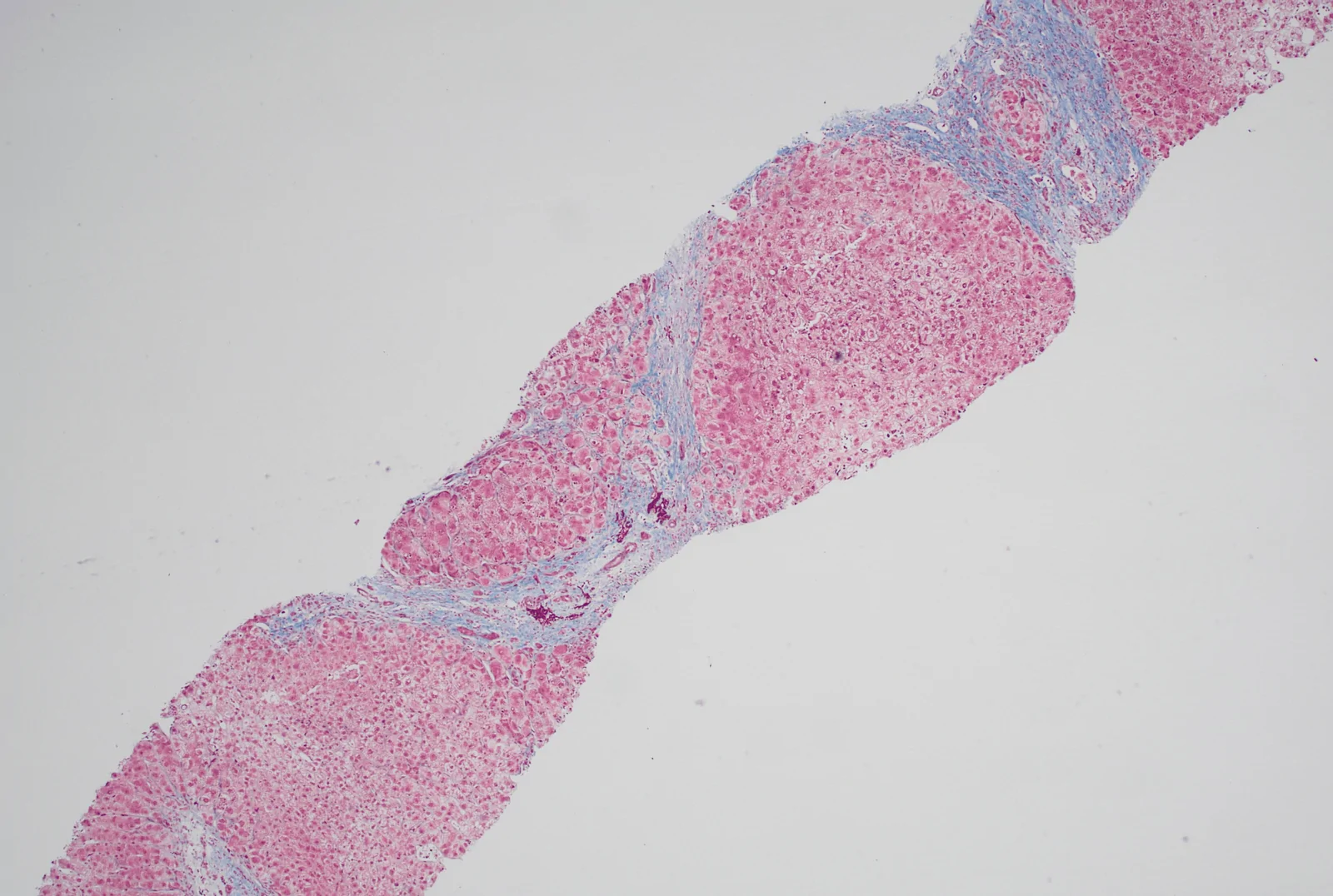
Masson's trichrome stain of the liver biopsy specimen. Bridging fibrous septa encircle regenerative hepatocyte nodules, consistent with established cirrhosis (40× magnification).

**Figure 2:**
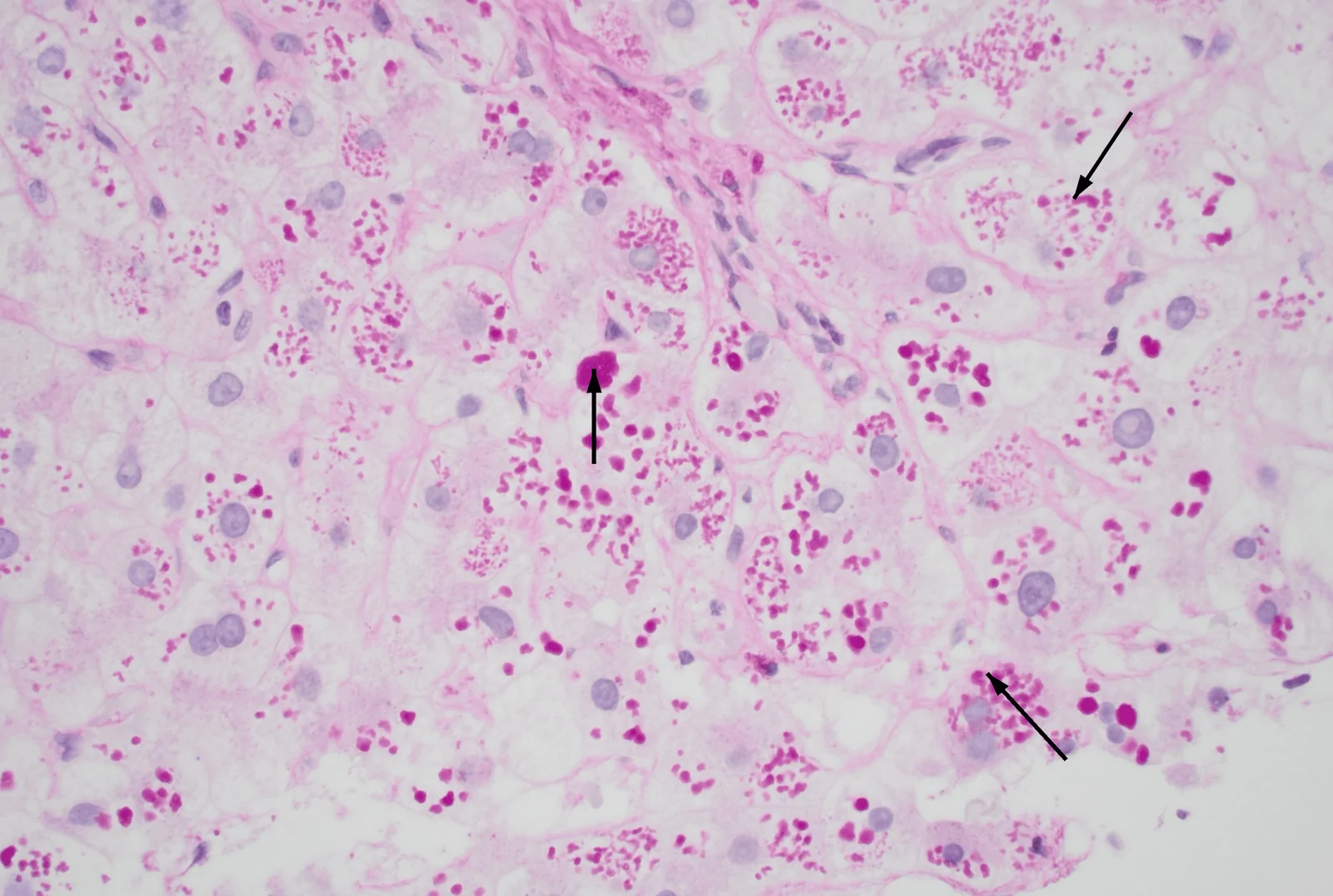
Periodic acid-Schiff-positive, diastase-resistant (PAS-D) stain of the liver biopsy specimen (400× magnification). Arrows indicate representative PAS-D intrahepatocytic globules within hepatocyte cytoplasm - the histologic hallmark of alpha-1 antitrypsin deficiency.

Sodium normalized to 133 mEq/L by hospital day nine. Jaundice had not improved at discharge, and she remained without ascites or asterixis. She was discharged in stable condition with referral to hepatology for evaluation, including consideration of transplantation given her MELD-Na of 32. Further outcome data were not available to the authors at the time of writing.

## Discussion

AATD is an inherited condition caused by a mutation in a single gene called SERPINA1. This deficiency has been associated with early-onset emphysema and liver cirrhosis [6]. It can present as cholestasis in infancy or chronic liver disease in childhood. Adults over the age of 50 are at risk for cirrhosis and hepatocellular carcinoma [1,7]. Liver disease results from hepatocellular retention of misfolded A1AT within the endoplasmic reticulum, driving inflammation and progressive fibrosis. These molecules persist as PAS-D globules, the histologic hallmark of the disease [1,8].

Part of the diagnostic evaluation includes testing a patient for their genotype of the SERPINA1 gene. PiZZ homozygotes occur in roughly 1:2000 to 1:3000 individuals of European ancestry [7]. Fibrosis was present in 20-38% of adults by non-invasive testing and about 35% in cross-sectional biopsy series in PiZZ homozygotes [9]. It is largely accepted that PiZZ homozygote individuals are at substantial risk for liver disease. PiMZ heterozygotes occur in up to 4% of individuals of European ancestry. Historically, this was labeled a benign finding, but now it is best understood as an aggravating cofactor for liver fibrosis if concurrent metabolic or environmental stressors, such as alcohol abuse, are present [10]. A case-control study in 2019 showed that PiMZ variants were present in 6.2% of alcohol misusers with cirrhosis but only in 2.2% of alcohol misusers without significant liver injury. Alcohol misusers with PiMZ had an OR for cirrhosis of 5.8 (95% CI 2.9-11.7) compared with alcohol misusers without PiMZ [3].

Serum levels of AAT reliably identify PiZZ homozygotes but cannot exclude heterozygosity. When evaluating for AATD, serum AAT alone cannot be relied upon, as serum levels in PiMZ heterozygotes may fall near the reference range and the liver phenotype tracks with mutant protein burden rather than serum deficiency [4]. PiMZ individuals carry roughly doubled adjusted odds of liver fibrosis or cirrhosis compared with non-carriers (aOR 1.7, 95% CI 1.2-2.2) despite only modestly reduced serum AAT concentrations [9]. Serum AAT level should be utilized as a screening tool, but genotyping should be performed if there is high suspicion.

Both genotypes produce the same lesion on liver biopsy - PAS-D positive, diastase-resistant intrahepatocytic globules of polymerized Z-AAT with associated portal inflammation and fibrosis - but PiMZ livers show far fewer inclusions and lower fibrosis stages [9]. Inclusion abundance rises with fibrosis stage, where all cirrhotic MZ patients had inclusions whereas most non-cirrhotic MZ patients had absent or rare inclusions [9,11].

Counseling varies between the two genotypes, as PiZZ carries a high absolute risk of both lung and liver disease, whereas PiMZ confers a low absolute risk that becomes significant in the presence of cofactors. Counseling for both genotypes includes smoking cessation, avoidance of environmental pollutants, alcohol moderation or abstinence, weight management, and vaccinations. Genetic testing of first-degree relatives is also recommended for both genotypes. PiZZ-specific counseling includes an annual liver panel, liver ultrasound, and transient elastography. There are no formal liver surveillance guidelines for heterozygotes. A suggested approach includes liver panel and non-invasive fibrosis assessment in those with cofactors such as obesity, alcohol use, and diabetes [7]. Heterozygous PiMZ carriage should no longer be regarded as clinically silent.

## Conclusions

This case illustrates that PiMZ heterozygosity is not a benign incidental finding but a clinically relevant cofactor that can compound the effects of alcohol use in the progression to decompensated cirrhosis. Because serum AAT levels may fall within or near the normal range in PiMZ carriers, genotyping should be considered in patients with cirrhosis of uncertain or multifactorial etiology, particularly when alcohol use or other metabolic stressors are also present. Greater clinician awareness of this genotype-phenotype relationship may improve diagnostic clarity and support more individualized counseling and surveillance for at-risk patients.

## References

[1] American Journal of Respiratory and Critical Care Medicine (2003). American Thoracic Society/European Respiratory Society statement: standards for the diagnosis and management of individuals with alpha-1 antitrypsin deficiency. Am J Respir Crit Care Med.

[2] Schneider CV, Hamesch K, Gross A (2020). Liver phenotypes of European adults heterozygous or homozygous for Pi∗Z variant of AAT (Pi∗MZ vs Pi∗ZZ genotype) and noncarriers. Gastroenterology.

[3] Strnad P, Buch S, Hamesch K (2019). Heterozygous carriage of the alpha1-antitrypsin Pi*Z variant increases the risk to develop liver cirrhosis. Gut.

[4] Marek GW, Malhi H (2026). Protein aggregates in heterozygous alpha-1 antitrypsin phenotype is a marker for progressive disease. Gastro Hep Adv.

[5] Adrogué HJ, Tucker BM, Madias NE (2022). Diagnosis and management of hyponatremia: a review. JAMA.

[6] World Health Organization (1997). Alpha 1-antitrypsin deficiency: memorandum from a WHO meeting. Bull World Health Organ.

[7] Strnad P, McElvaney NG, Lomas DA (2020). Alpha1-antitrypsin deficiency. N Engl J Med.

[8] Carrell RW, Lomas DA (2002). Alpha1-antitrypsin deficiency — a model for conformational diseases. N Engl J Med.

[9] Fromme M, Schneider CV, Pereira V (2022). Hepatobiliary phenotypes of adults with alpha-1 antitrypsin deficiency. Gut.

[10] Goltz D, Hittetiya K, Vössing LM, Kirfel J, Spengler U, Fischer HP (2014). α₁-antitrypsin PiMZ heterozygosity has an independent aggravating effect on liver fibrosis in alcoholic liver disease. Virchows Arch.

[11] Fischer HP, Ortiz-Pallardó ME, Ko Y, Esch C, Zhou H (2000). Chronic liver disease in heterozygous α1-antitrypsin deficiency PiZ. J Hepatol.

